# Thrombotic complications and tip position of transjugular chronic dialysis catheter scheduled into superior vena cava: Findings on HR-MRCP and HR-T_2_WI

**DOI:** 10.1097/MD.0000000000007135

**Published:** 2017-06-30

**Authors:** Whenzheng Li, Fang Li, He Wang, Xueying Long, Obin Ghimire, Yigang Pei, Xiangcheng Xiao, Jianping Ning

**Affiliations:** a Department of Radiology, Xiangya Hospital, Central South University, Changsha; b Philips Healthcare, Buiding, Shanghai; c Department of Nephrology, Xiangya Hospital, Central South University, Changsha, China.

**Keywords:** chronic dialysis catheter, HR-MRCP, HR-T_2_WI, thrombotic complications, tip position

## Abstract

**Background:**

Catheter-related thrombotic complications(TCs) can occur during the long term use of a chronic dialysis catheter (CDC), including fibrin sheath (FS), mural thrombosis (MT), venous thrombosis (VT), and intraluminal clots (IC), which has not been reported with MRI. The aim of our study was to evaluate the determination of catheter tip position (TP) and resolution of TCs in patients with transjugular CDC scheduled into the superior vena cava using high resolution magnetic resonance cholangiopancreatography (HR-MRCP) and T_2_-weighted imaging (HR-T_2_WI).

**Methods:**

The study protocol was approved by the local Research Ethics Committee. Informed consent was obtained from all patients. In total, 41 consecutively enrolled transjugular CDC patients with suspected catheter dysfunction were scanned with HRMRCP and HR-T_2_WI. The distance from the top to the tip of the catheter and the presence and nature of catheter TCs were assessed by 2 experienced radiologists. Chest x-ray was taken within 1 to 2 days and CDC was withdrawn within 3 to 10 days from those patients with TCs identified by HR-MRI.

**Results:**

A total of 38 subjects successfully underwent HR-MRI, including 13 normal and 25 with TCs (fibrin sheath [FS]: n = 21, mural thrombosis [MT]: n = 7, venous thrombosis [VT]: n = 3, intraluminal clots [IC]: n = 4). There was no significant difference between HR-MRCP and chest x-ray in catheter TP determination (*P* = .124). Normal catheter appeared as “double eyes” on HR-T_2_WI and “double tracks” on HR-MRCP. TCs appeared as follows: FS displayed as a “thin ring” (<1mm) around the catheter, MT as patchy hyperintensity and VT as a “thick ring” (>5mm) on HR-T_2_WI. Unilateral IC appeared as a “single eye” on HR-T_2_WI and a “single track” on HR-MRCP (n = 3). Bilateral IC appeared as neither “eye” nor “track” (n = 1). Catheter withdrawal confirmed FS (n = 16), MT (n = 6), VT (n = 1), and IC (n = 4).

**Conclusion:**

HR-MRCP and HR-T_2_WI are promising methods for visualizing TP and TCs in CDC patients, and are helpful in adjusting the treatment plan and avoiding the risk of pulmonary embolism.

## 1 Introduction

For a dialysis patient with end-stage renal disease under routine dialysis, a well-functioning vascular access is essential for an efficient hemodialysis procedure. An arteriovenous fistula is known to be the best blood access due to the possibility of long-term use and the low-level of complications.^[1]^ However, for those patients who are not a good candidate for an arteriovenous fistula or those who require dialysis during maturation of the arteriovenous fistula, establishing an effective vessel access through the internal jugular vein is the best choice, with easy visualization of the jugular vein by ultrasound and direct connection to the superior vena cava and right atrium^[2]^ that is adequate to meet the dialysis requirement with the use a catheter of at least of 300cc per minute.^[1,3]^ In the United States, over 60% of patients begin hemodialysis with placement of transjugular chronic dialysis catheter (CDC).^[4]^ In China, the number of patients receiving CDC hemodialysis increases.

However, several complications can occur during the long-term use of a CDC, including sepsis, extravasations of infusions, pneumothorax, kinking, and thrombotic occlusion of the catheter, which can increase associated morbidity and mortality.^[5]^ It is, therefore, crucial to diagnose these thrombotic occlusions effectively and to determine the cause and type of thrombotic complications (TCs). Types of thrombotic complications, which can occur separately or in combination, include fibrin sheath (FS) around the catheter, intraluminal clots (IC) inside the catheter, mural thrombosis (MT) adhered to the venous wall, and venous thrombosis (VT) completely blocking the vein.^[5,6]^

For these catheter-related thrombotic occlusions, multislice spiral computed tomography venography (MSCTV) can provide excellent depiction of MT and VT,^[7]^ but cannot differentiate FS, or IC or distinctly display the double lumen of the catheter due to artifacts caused by barium elements in the catheter.^[8]^ In addition, MSCTV can cause contrast-induced nephropathy due to the use of contrast medium, especially in end-stage renal disease patients. To the best of our knowledge, visualization of these TCs by MRI has not been reported. Therefore, our purpose is to demonstrate the performance of high-resolution MRI (HR-MRI) without contrast medium in displaying CDC's tip position (TP) and in the detection and differentiation of correlative TCs.

Currently, the HR-MRI technique is an increasingly essential method in the precision medicine era. In our study, to avoid blood interference and clearly present TCs, we used high-resolution T_2_WI with turbo spin echo sequence (HR-T_2_WI) giving an empty appearance to the lumen of the catheter due to the relatively long echo time. High-resolution T2-weighted MRCP (HR-MRCP) with 3D-SPACE (3-dimensional sampling perfection with application optimized contrast using different flip angle evolutions) was used to display the catheter double lumen, TP and any possible IC, which depend on the slow attenuation characteristic of water.

Chest x-ray cannot display any TC types, However, it can clearly visualize the catheter because the latter contains barium elements.^[8]^ In addition, have maintained that chest x-ray is recommended as the first-line method for locating the catheter's TP.^[9,10]^ Venugopal et al^[11]^ also considered that chest x-ray was the gold standard for identifying catheter tip malposition. Therefore, in our study, chest x-ray was used as the reference standard for assessment of the tip location on HR-MRCP by measuring the distance from the top to the tip of the catheter.

## 2 Methods

### 2.1 Patients

This prospective study was approved by the institutional review board. Written informed consent was obtained from all participants prior to examination. From April 2014 to August 2015, all patients with CDC transjugular access and suspicion of catheter dysfunction (failure to attain a sufficient extracorporeal blood flow of ≥300 mL/min with a prepump arterial pressure more negative than −250 mm Hg for 2 weeks)^[3,12]^ were consecutively recruited and underwent MRI and chest x-ray in our study.

A total of 41 subjects including 17 males and 24 females (mean age, 62.4 ± 14.2 years) underwent 1.5T MRI to identify the catheter TP and possible presence of TCs.

### 2.2 MRI

Before MRI, all transjugular tunneled dual-lumen CDC (size: 14.5F, Covidien llc, 15 Hampshire Street Mansfield, MA ) subjects were pulled out the previous installed heparin and then injected 5 mL physiological saline into each lumen. The injection was stopped at the moment to feeling appreciable resistance in the operating process. The patient was then examined using a standard clinical radiology suite with a 1.5T Magnetom Area MRI (Siemens Healthcare, Erlangen, Germany) equipped with a manufacturer's 20-channel head coil combined with a dedicated abdominal18-channel body phased-array coil. First, 3-plane localizer was performed for neck and chest, followed by coronal and sagittal T_2_-weighted True FISP (TR/TE, 39.2/1.2; slice thickness, 4 mm; slice gap, 0.8 mm) and axial T2-weighted haste sequence (TR/TE, 700/87; slice thickness, 6 mm; slice gap, 0.6 mm). HR-MRCP imaging with 3D SPACE was then obtained using a 3-dimensional navigator-triggered technique and HR-T_2_WI with 2D turbo spin echo was gained with a peripheral pulse wave gated technique under multiple breath-holds to display the catheter tip and any associated TCs. The scanning parameters are presented in Table 1. Finally, the axial T_1_-weighted vibe sequence (TR/TE, 3.47/1.27; slice thickness, 3 mm; slice gap, 1 mm) was performed. The total MRI data acquisition time was approximately 20 to 25 minutes for each patient.

**Table 1:**
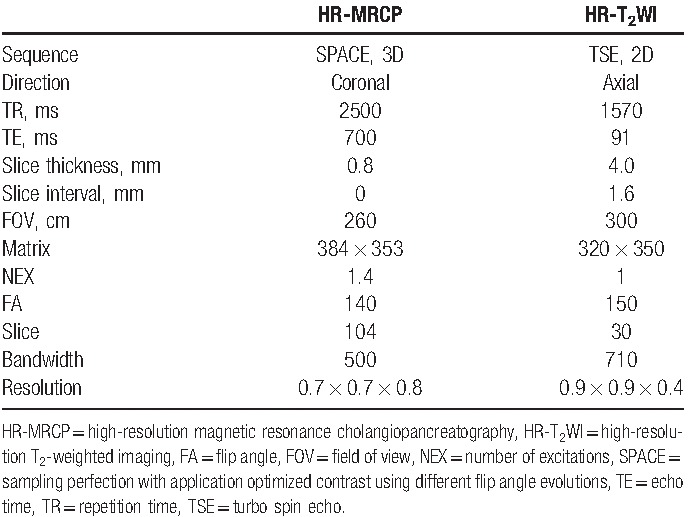
Scanning parameters for HR-MRCP and HR-T_2_WI.

### 2.3 Chest x-ray

Standard poster anterior chest x-rays were obtained within 1 to 2 days after MRI scan using standard digital radiographic equipment (Axiom Aristos MX, Siemens Medical Systems, Forchheim, Germany) and storage phosphor plates (Kodak PQ Elite CR direct view, Carestream Health Inc., Rochester, NY) with the following parameters: tube current, 80 kV; tube–film distance, 1.2 m; and exposure time product, 3–5mAs.

### 2.4 Image analysis

All chest x-ray and MRI images were transmitted to an imaging workstation (Advantage Workstation 4.4, GE Healthcare, Buc, France) for each patient. For HR-MRCP images, maximum intensity projection was performed to show the TP and double-lumen of CDC.

**Figure 1:**
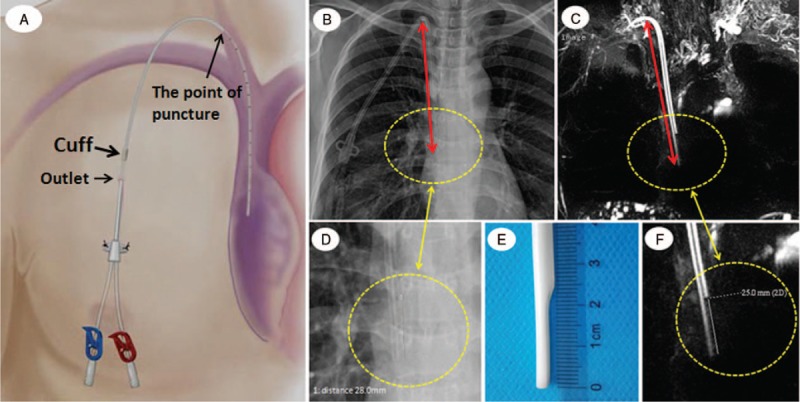
Schematic picture (A) and images of transjugular CDC insertion to illustrate the distance measurement technique for assessing the reliability of HR-MRCP in showing the catheter tip position and determining a relative magnification error on the chest x-ray (B, D) and HR-MRCP (C, F). Long double-headed arrows show distances from the top of catheter's inferior edge to the tip as shown in B (chest x-ray) and C (HR-MRCP). Circles in B–D and F indicate catheter tip locations. Panels D and F are magnified pictures of B and C, respectively. The photograph of catheter tip in D shows the measured distance between the staggered termini of the 2 catheter lumens in comparison with E (actual length) and F (HR-MRCP). CDC = chronic dialysis catheter, HR-MRCP = high-resolution magnetic resonance cholangiopancreatography.

The magnification error on chest x-ray and HR-MRCP images was estimated by measuring the distance between one lumen tip and the other lumen tip in 1 subject, and comparing it to the actual distance as illustrated in Figure 1. The tunneled dual-lumen catheter was passed through the skin at the outlet point, held in place by a cuff (long arrow, Fig. 1A), and inserted into the superior vena cava through the right internal jugular (point of puncture, Fig. 1A). The length of the catheter and the configuration were invariable from the cuff point to the tip. Although the cuff could not be visualized, the top and the tip of the catheter were clearly shown on chest x-ray and HR-MRCP. The distances from the top of the catheter's inferior edge to the tip were measured on both HR-MRCP and x-ray (long double-headed arrows, Fig. 1B [chest x-ray] and Fig. 1C [HR-MRCP]) and used to evaluate HR-MRCP reliability for showing catheter tip location relative to **c**hest x-ray. In addition, the lengths between one lumen tip and the other tip were measured on both HR-MRCP and x-ray (circles, Fig. 1B–D, F) and used to determine the relative magnification error between x-ray and HR-MRCP by comparing with the actual length (Fig. 1D). The magnification error (ME) was then calculated as ME = (distance on chest x-ray–distance on HR-MRCP)/distance on HR-MRCP.^[13]^ In our study, the length was 28 mm on x-ray, whereas the true length and the length on HR-MRCP was 25 mm, yielding a magnification error (ME) = (28–25)/25 = 0.12. The corrected length on x-ray = the measured distance on x-ray ÷ [1 + ME]) was then used as a reference standard for assessing the accuracy of the apparent tip location on HR-MRCP. The distance from the top point of the catheter's inferior edge to the tip of the catheter for each subject was measured on chest x-ray and HR-MRCP by the consensus of 2 experienced radiologists and the accuracy of tip location shown on HR-MRCP was then assessed by comparing the measured distance on HR-MRCP with the corrected distance on chest x-ray. Tip locations in superior vena cava and right atrium were regarded as normal positions to meet the dialysis requirement of 300cc per minute. Patients with unclear catheter's TP on chest x-ray and motion artifacts on HR-MRCP and HR-T_2_WI were excluded. Finally, the presence of IC and gas was predicted based on hypointensity in the catheter lumen on HR-MRCP.

**Figure 2:**
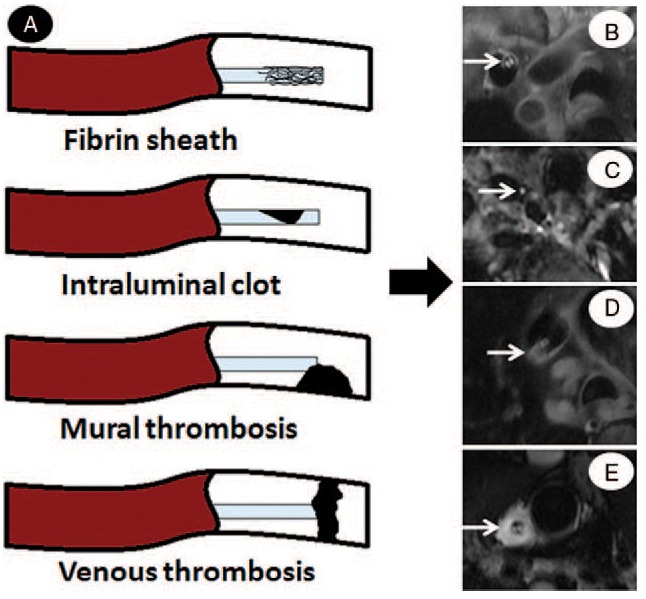
Schematic pictures describing different thrombotic complications and their appearance on HR-T_2_WI. Complications illustrated in A include fibrin sheath, intraluminal clot, mural thrombosis, and venous thrombosis. Fibrin sheath appeared as a ring of hyperintensity (long arrow, B), surrounding the catheter (hypointensity) on HR-T_2_WI. The saline in the lumens showed hyperintensity like “2 eyes” on HR-T_2_WI. Intraluminal clots showed low signal with a “single eye” when the blood clots filled in 1 lumen (long arrow, C) and no “eye” sign when clots were in both lumens. Mural thrombosis appeared as patchy hyperintensity adhered to the vessel wall without completely occluding the vein (long arrow, D). Venous thrombosis appeared as a thick ring of high signal on HR-T_2_WI (long arrow, E) and occluded the whole vein. HR-T_2_WI = high-resolution T_2_-weighted imaging.

IC was assessed by HR-T_2_WI combined with HR-MRCP, and the other TCs (FS, MT, and VT) were evaluated by HR-T_2_WI with the consensus of 2 experienced radiologists for each subject. Schematic pictures are shown in Figure 2. For those patients with TCs evaluated by HR-MRCP and HR-T_2_WI, CDCs were removed within 3 to 10 days after MRI. Patients without TCs revealed by HR-MRCP combined with HR-T_2_WI were continued on dialysis after adjusting the catheter tip location and direction. The evaluation of TCs on HR-MRI and the measurement of distances on HR-MRCP and on chest x-ray were performed by 2 radiologists with a blinded and randomized reading.

### 2.5 Statistical analysis

A paired-samples *t*-test was used to compare the mean differences between measurement data groups. *P* > .05 was considered to indicate no significant difference. In addition, mean ± standard deviation (SD) was used in measurement data and constituent ratio was used in count data. All statistical analyses were performed by using commercially available software (SPSS for Windows, version 13.0; SPSS, Chicago, IL).

## 3 Results

A total of 38 CDC patients’ images were evaluated after successfully performing x-ray, HR-MRCP, and HR-T_2_WI. Three patients’ images were excluded, 1 due to motion artifacts from irregular respiratory motion, and 2 due to intravenous artifacts from the inexhaustive flowing empty effect. In the 38 subjects, the reasons for renal function failure were as follows: chronic glomerulonephritis (n = 16), diabetic nephropathy (n = 10), hypertensive nephropathy (n = 5), polycystic kidney disease (n = 3), rapidly progressive glomerulonephritis (n = 2), ANCA (anti-neutrophil cytoplasmic antibody) associated systemic vasculitis (n = 1), and nephrectomy for tumor in a solitary kidney (n = 1). The duration time of CDC placement was 1 to 48 months and the median time was 11.5 months

**Figure 3:**
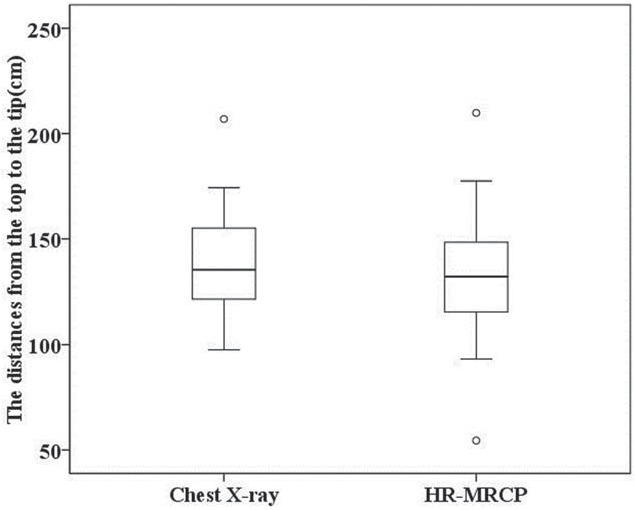
Box plot of the distances’ distribution from the top to the tip on chest x-ray and HR-MRCP. The figure was displayed a normal distribution in the distances between chest x-ray and HR-MRCP. HR-MRCP = high-resolution magnetic resonance cholangiopancreatography.

**Figure 4:**
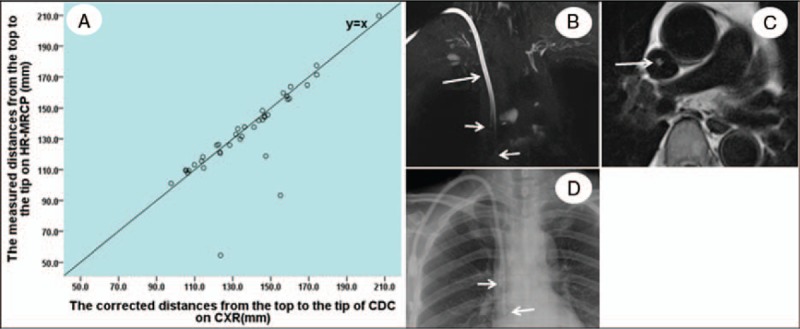
(A) Scatter plot of the distances measured from top to tip of catheter on HR-MRCP versus chest x-ray. (B–D) An example scan showing no complications with normal findings on HR-MRCP and HR-T_2_WI performed in a 56-year-old end-stage renal disease patient catheterized for 23 months. Note the double lumens of the catheter appearing as hyperintensity in a “double track” sign on HR-MRCP (long arrow, B) and as “double-eyes” sign on HR-T_2_WI (long arrow, C). Catheter tips presented distinctly on HR-MRCP (measured distance: 131.7 cm) and x-ray (corrected distance: 132.9 cm) and there was no significant difference between them (B and D, short arrows). HR-MRCP = high-resolution magnetic resonance cholangiopancreatography, HR-T_2_WI = high-resolution T_2_-weighted imaging.

In comparing HR-MRCP with chest x-ray, the findings of catheter length from the top to the tip are shown in Table 2 and Figure 3. There was no significant difference in the mean catheter length derived from HR-MRCP and x-ray (*P* = .124) (Table 2, Fig. 4), although the lengths were anomalously short on HR-MRCP compared to x-ray in 3 subjects (Fig. 4A). In addition, 7 patients had abnormal catheter tip locations as shown by HR-MRCP (Table 3). The accuracy of these determinations was shown to be 100% in all cases based on x-ray. On HR-MRCP, the double lumen catheter structure was clearly displayed in these 38 subjects (Fig. 4B and C). In addition, intraluminal gas was found at the top of the catheter in 2 subjects.

**Table 2:**
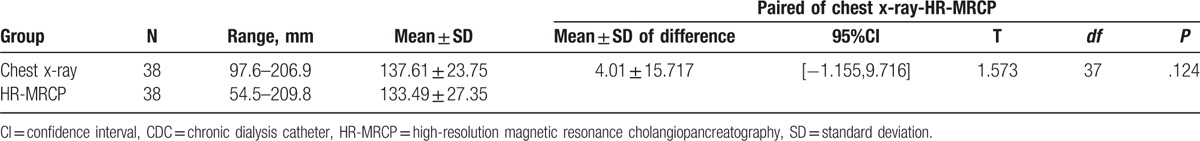
Distance from catheter's top to tip on HR-MRCP and x-ray and CDC's tip location on HR-MRCP compared to x-ray by the 2 sample paired test.

**Table 3:**
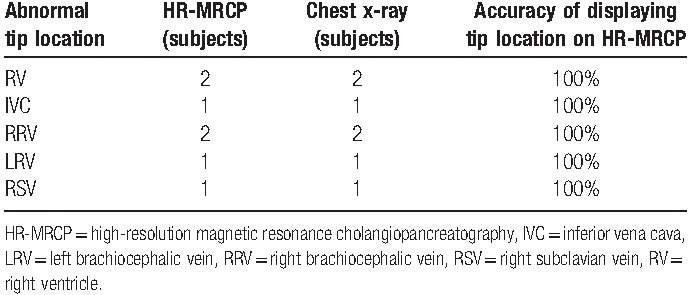
Abnormal tip location findings on HR-MRCP confirmed by **c**hest x-ray.

**Figure 5:**
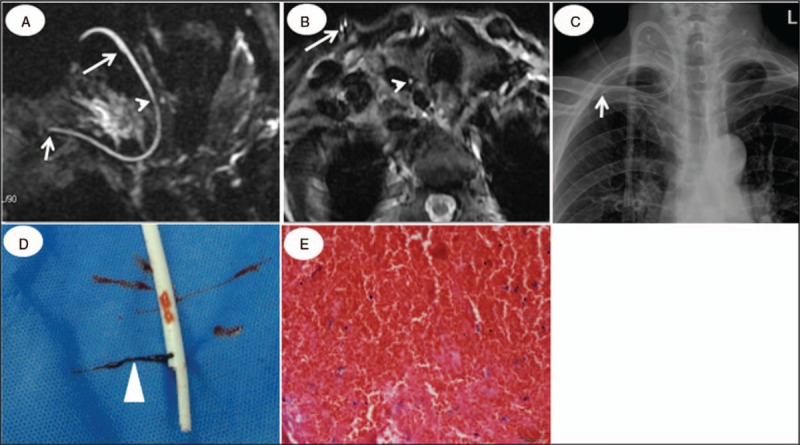
A case with unilateral intraluminal clots and tip location abnormity in a 62-year-old end-stage renal disease patient catheterized for 3 months. The catheter tip was displayed in the right subclavian vein on HR-MRCP, confirmed by the chest x-ray (short arrow, A and C). The “Single track” sign on HR-MRCP and “single eye” on HR-T_2_WI in the distal catheter (arrowhead, A, B) indicate clot formation in the other lumen, confirmed after catheter removal (triangle, D). Panel E shows clots composition: large number of red blood cells and few platelets (H&E, ×40 magnification, E). Note the normal “double track” sign on HR-MRCP and the “double-eyes” sign on HR-T_2_WI in the proximal catheter (long arrow, A, B). H&E = hematoxylin and eosin, HR-MRCP = high-resolution magnetic resonance cholangiopancreatography, HR-T_2_WI = high-resolution T_2_-weighted imaging.

**Figure 6:**
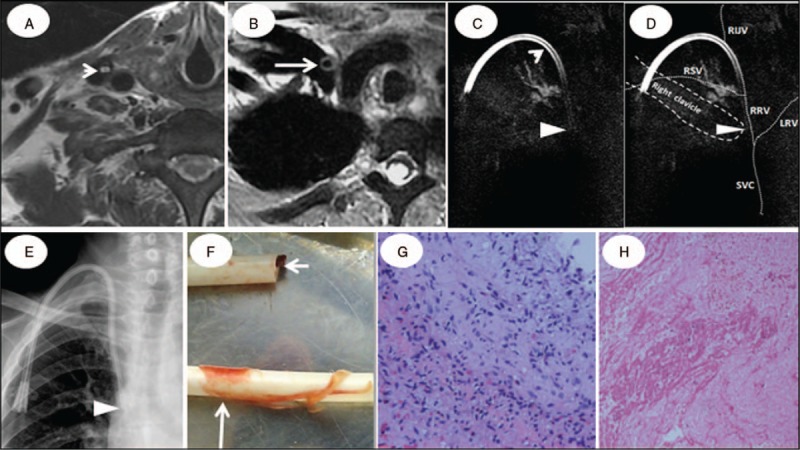
A case with fibrin sheath and dual intramural clots in a 68-year-old end-stage renal disease patient catheterized for 38 months. The normal “double-eyes” sign was shown by HR-T_2_WI and the “double-track” sign on HR-MRCP from the level of the internal jugular vein to the level of right proximal brachiocephalic vein (arrow, A,C). The absence of any “eye” sign on HR-T_2_WI or the “track” sign on HR-MRCP from the level of right proximal brachiocephalic vein to the level of superior vena cava indicates bilateral intraluminal clots (Fig. B–D). Panel D is a modified schematic diagram of the anatomical location of veins and catheter in C. Clots were verified by the withdrawn catheter (F, short arrow) and consisted of abundant red blood cells and few platelets (H&E, ×40 magnification, H). Note obviously shorter distance from top to tip on HR-MRCP than on the x-ray (54.4 cm vs123.6 cm; compare triangle: tip location, C, E). A “thin ring” signal surrounding the catheter in the right brachiocephalic vein at distal level (long arrow, B) hinted fibrin sheath, confirmed by the withdrawn catheter (long arrow, F), and composed of many fibroblasts and a few foam cells (H&E, × 40 magnification, G). H&E = hematoxylin and eosin, HR-MRCP = high-resolution magnetic resonance cholangiopancreatography, HR-T_2_WI = high-resolution T_2_-weighted imaging.

In our study, TCs were not found in 13 subjects that displayed normal hyperintensity with “double-eyes” sign on HR-T_2_WI and “double track” sign on HR-MRCP (Fig. 4B and C). For thrombotic catheter complications, the “single track” sign on HR-MRCP and “single eye” on HR-T_2_WI were found when IC happened in 1 lumen (Fig. 5), and “track” sign on HR-MRCP and “eye” sign on HR-T_2_WI could not be shown bilaterally at the level of the clot (Fig. 6). In addition, FS appeared as “thin ring” sign (<1 mm) surrounding the catheter (Fig. 6), MT showed patchy hyperintensity and VT presented as a “thick ring” (>5 mm) on HR-T_2_WI.

Among 25 subjects with TCs identified by HR-MRCP combined with HR-T_2_WI, there were 21 patients with FS (55.3%), 7 with MT (15.8%), 3 with VT (7.9%), and 4 with IC (10.5%) (Table 4). TCs were confirmed after the removal of catheter in 21 patients, yielding findings of FS (n = 16), MT (n = 6), VT (n = 1), and IC (n = 4) (Table 4). As shown in Table 4, the negative findings of different types of TCs on HR-MRCP and HR-T_2_WI were confirmed after removal of the catheter, with the exception of 1 case of a false negative where removal of the catheter revealed FS. In these 38 subjects, only 6 patients had dialysis stopped to withdraw heparin or injected saline when feeling resistance in 1 lumen (confirmed as 3 FS and 3unilateral IC), and 1 subject was occluded in both lumens (confirmed as a bilateral IC) before the MRI scan. Unfortunately, 2 patients died due to pulmonary embolism after withdrawal of the catheter.

**Table 4:**
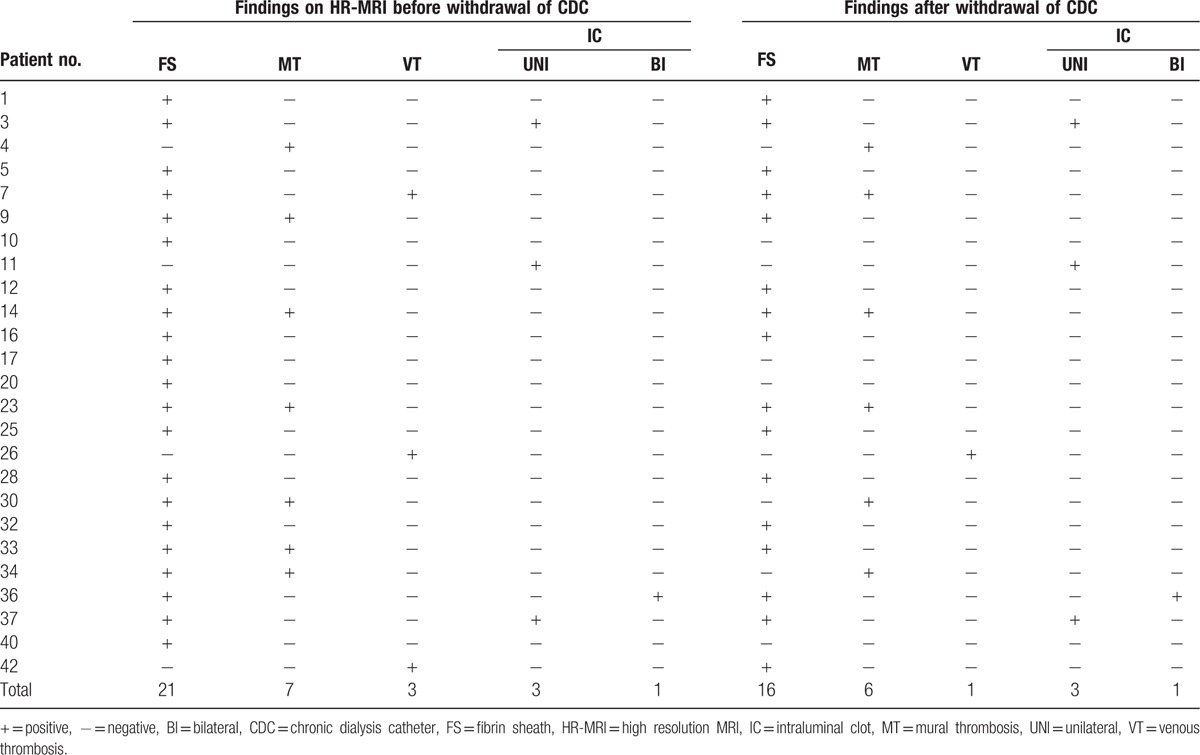
Types of thrombotic complications identified on HR-MRI and after withdrawal of CDC.

## 4 Discussion

Recently, many types of catheter have been inserted into the superior vena cava or the right atrium, including dialysis catheters, peripherally inserted central catheters, and central venous catheters, whose purposes are to establish vascular access, administer therapy, and improve the quality of life.^[5]^ However, TCs can arise from catheter placement, especially for CDC, due to long-term emplacement and the relatively large size of the catheter. These complications include FS, IC, MT, and VT,^[5,6]^ and might lead to catheter dysfunction and even pulmonary embolism. Therefore, it is crucial to have precise imaging of these complications with MRI, as it is beneficial in decreasing the occurrence of pulmonary embolism and adjusting the treatment plan. Accurate identification of IC requires accurate display of the catheter's tip location on HR-MRCP. Therefore, in our study, HR-MRCP was used to determine the tip position. HR-T_2_WI was used to display FS, MT, and VT, whereas HR-MRCP combined with HR-T_2_WI were used to identify IC.

The venous blood contains paramagnetic deoxyhemoglobin and has a short T2 relaxation time in comparison with water.^[14]^ This can cause T2-weighted signals to decrease due to the relatively short T2 and the local magnetic field asymmetry^[15]^ in a small lumen. Thus, the motionless blood in catheters presents hypointensity on HR-MRCP and HR-T_2_WI. Prior to MRI, 5 mL 0.9% saline was infused into each catheter lumen to prolong the T2 relaxation time and aid in locating the catheter tip, detecting IC, and identifying the double lumen structure on HR-MRCP and HR-T_2_WI. In our study, we were able to infuse saline into 1 or both catheter lumens in 37 patients with recognizable tip positions on HR-MRCP. However, there were 3 patients for whom the distance from the top to the tip on HR-MRCP was markedly less than on chest x-ray. In 1 subject with a bilateral IC, the discrepancy was due to less water content than venous blood. For the other 2 patients, the shorter apparent distance may have resulted from backflow of venous blood into the catheter after saline injection, leading to the signal loss on HR-MRCP due to the shorter T2 of venous blood. Thus, it is difficult to differentiate IC from intraluminal blood, especially in the tip of the catheter. However, IC should be suspected if there is resistance when injecting saline, with intraluminal venous blood being more likely when there is no resistance.

Nevertheless, comparing the average distance from the top to the tip of catheter in all 38 patients, there was no significant difference between HR-MRCP and chest x-ray (*P* = .124), indicating that HR-MRCP is a reliable method for visualizing the catheter tip location. Hence, HR-MRCP might be an alternative to chest x-ray for delineating TP without any radiation. What is more, it is very essential to determine the presence of clots in 1 or both catheter lumens. There is a distinct advantage in the ability of HR-MRCP to detect IC as compared to x-ray. In our study, 7 patients presented with abnormal positions on HR-MRCP, with the accuracy of these determination being confirmed in all cases by chest x-ray.

In our research, a normal, non-occluded catheter appeared as hyperintensity in the form of “double tracks” sign on HR-MRCP and “double-eyes” on HR-T_2_WI, due to saline filling the 2 catheter lumens. An IC will present with more hypointensity than venous blood, because it contains less water and has a shorter T2 relaxation time. Therefore, a blood clot in 1 lumen appeared as a “single track” on HR-MRCP and “single eye” on HR-T_2_WI, and the absence of any “track” sign on HR-MRCP and “eye” sign on HR-T_2_WI indicated clots in both lumens. In our results, IC were found on HR-MRCP and HR-T_2_WI in 4 subjects, located near the tip and completely obstructing 1 or both lumens of the catheter. Such clots result from the coagulation cascade. They consist of abundant red blood cells and a few platelets^[16]^ and account for 5% to 25% of all catheter occlusions.^[5]^ Intraluminal gas also presents with no signal due to the short T2 relaxation time and will also appear as a “single track” on HR-MRCP and “single eye” on HR-T_2_WI. However, intraluminal gas is only located at the top of the catheter because it is the highest position when the patients lying on the MRI table, and therefore the position of the hypointensity in the catheter is a vital differentiation between gas and IC.

Catheter-associated FS is the commonest reason for CDC failure and can be composed of thrombus, fibroblasts, endothelial cells, and collagen forming a layer about 1 mm thick around the outside of the catheter. In our study, the thickness of the FS was less than 1 mm on HR-T_2_WI in all 21 subjects identified with this TC.^[17]^ The sheath covers the inlet and outlet holes of a catheter, acting as a 1-way valve, interfering with the catheter function and preventing effective hemodialysis.^[16]^ Oguzkurt et al^[18]^ found that FS formation was seen in up to 76% of short- or long-term central venous catheters by pull-back venography. Shanaah et al^[19]^ maintained that FS incidence was about 47%. In our data, FS incidence was 55.3% and presented with high signal like a “thin ring” (<1 mm) surrounding the catheter. The reason for hyperintensity may be due to extensive edema in patients with FS.^[20]^

Catheter-related thrombosis is a relatively common complication in end-stage renal disease patients with CDC, and includes VT and MT, as a result from coagulation cascade activation and platelet aggregation on the side of a vessel.^[21]^ Most of these thromboses occur within the first 100 days after catheter insertion.^[22]^ VT refers to a thrombus that develops near the catheter and occludes the vein. MT is a blood clot that adheres to the vessel wall and can occlude the catheter tip, but does not completely occlude the vein.^[5]^ Most patients with thrombosis are asymptomatic. Niers et al^[23]^ found that approximately 14% to 18% of patients have evidence of thrombosis without clinical symptoms. Symptomatic thrombosis occurs much less frequently, in approximately 5% of cases or less.^[24]^ In our 41 patients with suspected catheter-related complications, 10 cases of thrombus were found on HR-T_2_WI, appearing as patchy hyperintensity in the case of MT in 7 patients, and as a “thick ring” (>5 mm) for VT in 3 subjects.

Except for 1 false negative FS patient, the negative findings on HR-MRCP and HR-T_2_WI in our study were confirmed when the catheter was withdrawn. The single false negative was a case where the FS was completely obscured on the scan by a mural thrombosis filling the whole vein. The fibrin sheath was revealed because it adhered to the catheter, whereas the mural thrombosis did not. Our experiences thus show that HR-MRI is reliable to assess patients without catheter-related thromboses and can be safely used to adjust the catheter TP and direction. In our study, 13 catheters were adjusted after HR-MRI revealed no thrombotic complications, and dialysis through them was continued. Diagnosis of TCs in CDC patients by HR-MRCP combined with HR-T_2_WI was superior to surgical withdrawal because some thromboses cannot be withdrawn after the removal of catheter.

Effective management is needed to improve the survival and quality of life for CDC patients with TCs.^[25,26]^ For MT, VT, and IC, thrombolysis with urokinase or recombinant tissue plasminogen activator (rTPA) can be undertaken to restore adequate blood flow in most patients.^[27,28]^ For FS, catheter exchange should be performed to continue dialysis through interventional treatments.^[28]^ Therefore, in our view, it is very important to precisely evaluate the TCs’ type by HR-MRI, which cannot all be classified by MSCTV. Unfortunately, in 25 TCs subjects, 2 patients died due to FS and thrombus ultimately causing pulmonary embolism with catheter removal after treatment.

There are several limitations in our study. First, HR-MRCP is not always successful due to the interference of venous blood. The need to exclude venous blood from the catheter means that saline injection is necessary for HR-MRCP imaging. Second, the TCs’ size and location on HR-MRCP and HR-T_2_WI could not always be confirmed upon the surgical catheter's withdrawal. Third, in a few cases, some artifacts appeared on HR-T_2_WI due to the inexhaustive flowing empty effect. In addition, our study did not include other catheter types than CDC, such as peripherally inserted central catheters, temporary dialysis catheters, and central venous catheters.

In conclusion, end-stage renal disease patients with CDC placed via the jugular vein can develop several types of TCs, which can occur separately or in combination. HR-T_2_WI combined with HR-MRCP is a safe, non-invasive, relatively inexpensive, and reliable diagnostic method for both visualizing the position of the catheter tip and identifying related complications in these patients. These MRI techniques do not use gadolinium contrast or expose the patients to radiation as in CT scan. The resulting ability to more effectively differentiate the different types of thrombotic complications is helpful in avoiding pulmonary embolism and adjusting the management plan for the patient.

## Acknowledgments

I would like to thank Chen Jinbao, a statistics expert who helped us to finish the statistics findings in our research.

## References

[1] Beigi AA Sharifi A Gaheri H Placement of long-term hemodialysis catheter (permcath) in patients with end-stage renal disease through external jugular vein. Adv Biomed Res 2014;3:252. 25590030 10.4103/2277-9175.146381 PMC4283247

[2] Santoro D Benedetto F Mondello P Vascular access for hemodialysis: current perspectives. Int J Nephrol Renovasc Dis 2014;7:281–94. 25045278 10.2147/IJNRD.S46643 PMC4099194

[3] Griffiths RI Newsome BB Leung G Impact of hemodialysis catheter dysfunction on dialysis and other medical services: an observational cohort study. Int J Nephrol 2012;2012:673954. 22518313 10.1155/2012/673954 PMC3299278

[4] Ash SR Fluid mechanics and clinical success of central venous catheters for dialysis--answers to simple but persisting problems. Semin Dial 2007;20:237–56. 17555490 10.1111/j.1525-139X.2007.00284.x

[5] Baskin JL Pui CH Reiss U Management of occlusion and thrombosis associated with long-term indwelling central venous catheters. Lancet 2009;374:159–69. 19595350 10.1016/S0140-6736(09)60220-8 PMC2814365

[6] Rosovsky RP Kuter DJ Catheter-related thrombosis in cancer patients: pathophysiology, diagnosis, and management. Hematol Oncol Clin North Am 2005;19:183–202. 15639113 10.1016/j.hoc.2004.09.007

[7] Diaz ML Villanueva A Herraiz MJ Computed tomographic appearance of chest ports and catheters: a pictorial review for noninterventional radiologists. Curr Probl Diagn Radiol 2009;38:99–110. 19298909 10.1067/j.cpradiol.2008.05.002

[8] Ash SR Fluid mechanics and clinical success of central venous catheters for dialysis--answers to simple but persisting problems. Semin Dial 2007;20:237–56. 17555490 10.1111/j.1525-139X.2007.00284.x

[9] Barnacle A Arthurs OJ Roebuck D Malfunctioning central venous catheters in children: a diagnostic approach. Pediatr Radiol 2008;38:363–78. 486–487. 17932667 10.1007/s00247-007-0610-2 PMC2292495

[10] Zadeh MK Shirvani A The role of routine chest radiography for detecting complications after central venous catheter insertion. Saudi J Kidney Dis Transpl 2014;25:1011–6. 25193899 10.4103/1319-2442.139895

[11] Venugopal AN Koshy RC Koshy SM Role of chest X-ray in citing central venous catheter tip: a few case reports with a brief review of the literature. J Anaesthesiol Clin Pharmacol 2013;29:397–400. 24106371 10.4103/0970-9185.117114 PMC3788245

[12] Vascular Access Work Group. Clinical practice guidelines for vascular access. Am J Kidney Dis 2006;48 (suppl 1):S248–73. 16813991 10.1053/j.ajkd.2006.04.040

[13] Shigematsu H Koizumi M Yoneda M Magnification error in digital radiographs of the cervical spine against magnetic resonance imaging measurements. Asian Spine J 2013;7:267–72. 24353842 10.4184/asj.2013.7.4.267 PMC3863651

[14] Chavhan GB Babyn PS Thomas B Principles, techniques, and applications of T2^∗^-based MR imaging and its special applications. Radiographics 2009;29:1433–49. 19755604 10.1148/rg.295095034 PMC2799958

[15] Nagamine K Shimomura K Miyadera H Hemoglobin magnetism in aqueous solution probed by muon spin relaxation and future applications to brain research. Proc Jpn Acad Ser B Phys Biol Sci 2007;83:120–6. 10.2183/pjab.83.120 PMC3756736 24019590

[16] Singh SK Common A Perl J Peritoneal dialysis catheter malfunction because of encasement by an extraluminal fibrin sheath. Perit Dial Int 2012;32:218–20. 22383724 10.3747/pdi.2011.00172 PMC3525391

[17] Forauer AR Theoharis C Histologic changes in the human vein wall adjacent to indwelling central venous catheters. J Vasc Interv Radiol 2003;14:1163–8. 14514808 10.1097/01.rvi.0000086531.86489.4c

[18] Oguzkurt L Tercan F Torun D Impact of short-term hemodialysis catheters on the central veins: a catheter venographic study. Eur J Radiol 2004;52:293–9. 15544909 10.1016/j.ejrad.2003.12.004

[19] Shanaah A Brier M Dwyer A Fibrin sheath and its relation to subsequent events after tunneled dialysis catheter exchange. Semin Dial 2013;26:733–7. 23441925 10.1111/sdi.12074

[20] Mahadevan A Ramalingaiah AH Parthasarathy S Neuropathological correlate of the “concentric target sign” in MRI of HIV-associated cerebral toxoplasmosis. J Magn Reson Imaging 2013;38:488–95. 23440973 10.1002/jmri.24036 PMC4442780

[21] Chan MR Hemodialysis central venous catheter dysfunction. Semin Dial 2008;21:516–21. 19000124 10.1111/j.1525-139X.2008.00495.x

[22] Saber W Moua T Williams EC Risk factors for catheter-related thrombosis (CRT) in cancer patients: a patient-level data (IPD) meta-analysis of clinical trials and prospective studies. J Thromb Haemost 2011;9:312–9. 21040443 10.1111/j.1538-7836.2010.04126.x PMC4282796

[23] Niers TM Di Nisio M Klerk CP Prevention of catheter-related venous thrombosis with nadroparin in patients receiving chemotherapy for hematologic malignancies: a randomized, placebo-controlled study. J Thromb Haemost 2007;5:1878–82. 17723127 10.1111/j.1538-7836.2007.02660.x

[24] Lee AY Levine MN Butler G Incidence, risk factors, and outcomes of catheter-related thrombosis in adult patients with cancer. J Clin Oncol 2006;24:1404–8. 16549834 10.1200/JCO.2005.03.5600

[25] Mohamad AA Uhwut E Liew S Dialysis catheter fibrin sheath stripping: a useful technique after failed catheter exchange. Biomed Imaging Interv J 2012;8:e8. 22970064 10.2349/biij.8.1.e8 PMC3432227

[26] Major KM Bulic S Rowe VL Internal jugular, subclavian, and axillary deep venous thrombosis and the risk of pulmonary embolism. Vascular 2008;16:73–9. 18377835 10.2310/6670.2008.00019

[27] Sahin M Aktu RS Bulut M Percutaneous treatment of superior vena cava syndrome caused by chronic thrombosis. Turk Kardiyol Dern Ars 2014;42:76–9. 24481101 10.5543/tkda.2014.49207

[28] Gallieni M Giordano A Rossi U Optimization of dialysis catheter function. J Vasc Access 2016;17 (Suppl 1):42–6. 10.5301/jva.5000538 26951903

